# Evaluation of health-related quality of life before and after total hip arthroplasty in the elderly in Iran: a prospective cohort study

**DOI:** 10.1186/s40359-022-00762-3

**Published:** 2022-03-14

**Authors:** Aida Moarrefzadeh, Arash Sarveazad, Mehdi Mohammadpour, Mohammad Zareinejad, Mansour Bahardoust, Karim Pisoudeh, Sara Asgarian, Jebreil Shamseddin, Hasan toghraei Semiromi

**Affiliations:** 1 Department of Psychology, Master’s Degree Student of Psychology, Ahar Branch, Islamic Azad University, Ahar, Iran; 2 Colorectal Research Center, Iran University of Medical Sciences, Tehran, Iran; 3 Nursing Care Research Center, Iran University of Medical Sciences, Tehran, Iran; 4 Bone and Joint Reconstruction Research Center, Shafa Orthopedic Hospital, Iran University of Medical Sciences, Tehran, District 12, Mojahedin Islam St, Tehran, Iran; 5 Department of Epidemiology, School of Public Health, Shahid Beheshti University of Medical Sciences, Tehran, Iran; 6 Faculty of Medicine, Iran University of Medical Sciences, Tehran, Iran; 7 Infectious and Tropical Diseases Research Center, Hormozgan Health Institute, Hormozgan University of Medical Sciences, Bandar Abbas, Iran; 8 Minimally Invasive Surgery Research Center, Iran University of Medical Sciences, Tehran, Iran

**Keywords:** Quality of life, Total hip arthroplasty, Elderly

## Abstract

**Background:**

Owing to the direct impact of total hip arthroplasty (THA) on health-related quality of life (HRQOL) and the higher prevalence of THA in the elderly, this study aimed to compare HRQOL before, and after THA in the Iranian elderly.

**Methods:**

The present prospective cohort study was performed on 161 THA candidates. Demographic data were extracted from records of patients. Before, 6, and 12 months after THA, a Short Form 36 health survey (SF-36) was used to assess HRQOL. Before THA, 6 and 12 months after THA, Physical (PCS), and mental component scores (MCS) were obtained from a hundred separately for each subscale of the questionnaire. The Paired t-test was used to compare HRQOL before and after THA.

**Results:**

Both 6 and 12 months after THA, HRQOL was significantly increased compared to previous THA (*P* = 0.001). In the first half-year after THA, vitality and emotional state were not different from pre-surgery. However, 12 months after THA, these two subscales also were significantly improved. Although, 6 months after THA, the PCS has dramatically gone up compared to the previous THA (*P* = 0.012), despite MCS was remained steady. Nonetheless, by comparison with the before surgery, 12 months after THA, MSC notably improved (*P* = 0.048).

**Conclusion:**

HRQOL was appreciably improved by the THA in the elderly after 12 months. The improvement in HRQoL in the first 6 months after THA is related to the promotion in the physical aspect (PCS score), and in the second 6 months after THA is related to the promotion in the psychological aspect (MCS score).

**Supplementary Information:**

The online version contains supplementary material available at 10.1186/s40359-022-00762-3.

## Background

About a hundred years ago, the idea of performing surgery to treat osteoarthritis (OA) was materialized. Petersen has done total hip arthroplasty (THA) since 1938 via a cup was installed to cover the damaged head of the femur, which was a major innovation in THA at the time [1]. In the following years, by introducing the first prosthesis for THA, the surgery has entered a modern stage [2].

The number of THA is being increased gradually. Nowadays, 0.83% of the population, equivalent to 2.5 million people (1.4 million women and 1.1 million men) are undergone THA in the United States [3]. It is currently estimated that 400,000 THA are annually performed worldwide [4–6].

Such a noticeable growth in demand for THA showed that the positive effect on this surgery and surely, the Health-Related Quality Of Life (HRQOL) of patients. Although several studies around the world have assessed the effect of THA on HRQOL in OA patients worldwide [7–13]. The 36-item Short-Form(SF-36) Health Survey was the most popular of the questionnaires used to evaluate HRQOL, according to a systematic analysis by Pequeno et al. in 2020. [14]. This questionnaire examined eight aspects of HRQOL which included (1) physical and (2) social function, (3) physical and (4) emotional state, (5) mental health, (6) vitality/energy, (7) pain, and (8) perception of general health [15].

In addition to the positive effects, this surgery in terms of the postoperative complications can have adverse effects on HRQoL [16, 17]. Most studies on THA in Iran have examined the physical hip performance (by the Harris Hip Score) and pain (by the visual analog scale) after THA [18–20]. Among the few studies which have studied HRQOL, satisfactory results have reported after THA, we can mention the study of Sanei et al., in 2016. HRQOL was compared before and after THA. The results of the study have expressed that THA has considerably improved HRQOL in the patients with regard to developmental dysplasia of the hip. The middle-aged population (31 to 56 years, mean = 43.89) of the patients were studied [21]. Another Iranian study in this field was conducted in 2015 by Shahcheraghi et al. This retrospective study has examined the middle-aged population (22 to 75 years, mean = 48.83). The results of this study have showed the noticeable improvement in HRQOL after THA [22].

From the studies conducted in Iran, it can be seen that although THA is mainly performed in the elderly, but HRQoL related to THA in the middle-aged population was studied. The majority of population undergoing THA surgery are elderly, because the prevalence of this surgery increases dramatically with age. Kremers et al. showed that the proportions of THA in the United States were 0.58 percent in the 50 years old, 1.49 percent in the 60 years old, 3.25 percent in the 70 years old, 5.26 percent in the 80 years old, and 5.87 percent in the 90 years old since 2010 in the United States [3]. Considering few studies have compared HRQoL before and after THA in the elderly and such a study has not been performed in the Iranian population, this study aims to assess the indicators related to HRQoL before and after THA in the Iranian elderly.

## Methods

### Study setting and participants

Between September 2014 and September 2017, the current prospective cohort research was conducted on 161 THA candidate patients who had fractures and irreparable hip joint injury, according to the standard guideline Strengthening the Reporting of Observational Studies in Epidemiology (STROBE).Patients were identified bi-centrally from two high-volume arthroplasty hospitals in association with Iran University of Medical Sciences (IUMS), Tehran, Iran, by three orthopedic surgeons, and after applying the inclusion and exclusion criteria, they were admitted to the study consecutively. Under optimal conditions for this study, by evaluating the effect size of 0.31 in accord with the study of Bahardost et al. [10] by G Power software Version 3.1 estimated 65 people for the study. Sampling of the patients was performed by the availability sampling method. Regarding these two centers are specialized hip surgeries in Iran and patients are referred to these centers from almost all of Iran, nevertheless, this sample could be representative of the elderly population of Iran.

### Eligibility criteria

Inclusion criteria included age greater than 60 years, THA surgery was due to the femoral head fracture and OA lesions, filling out a personal consent form, and availability for postoperative follow-up. Exclusion criteria included conducting THA surgery due to accidents (since there was no disease in the hip joint before to the accident in these patients As a consequence, prior to THA, their HRQOL was unaffected), as were those with significant psychiatric illnesses (because in these patients before and after THA, HRQOL were affected by psychological reasons, not just because of THA).Patients with follow-ups were less than 6 months, and those who died during the study were excluded as well (because in these patients, the follow-up period was not completed, and data collection remained incomplete).

### Variables

Clinical and demographic data of patients included age, sex, body mass index (BMI), level of education, smoking, and co morbidities. Furthermore, main variables in the present study included physical (PCS) and mental component scores (MC).

### Data collection

Clinical and demographic data were extracted and recorded from patients' records. Before THA, 6 and 12 months after THA, the patient filled out a face-to-face quality of life assessment questionnaire. SF-36 was applied to assess HRQoL in this study (Additional file 1) [23]. The validity and reliability of this questionnaire (SF-36) in Iran were confirmed by Montazeri et al. in 2005 [24]. Scores were acquired from a hundred separately for each subscale of the questionnaire. A lower score demonstrated a worse situation in which in each of these subscales. Before THA, both 6 and 12 months after THA, PCS and MCS were calculated and recorded out of 100. PCS and MCS defined two aspects (physical and mental) of HRQOL [25].

### Statistical analysis

All patient data were analyzed by SPSS software version 22. The mean ± SD was used to report quantitative variables. Frequency (%) was used to report qualitative variables. The normality of the distribution of variables was determined via the Kolmogorov–Smirnov test. Considering the dependence of study groups, assuming the distribution of variables was normal, Health-related indicators and the average score of HRQoL were drawn an analogy before, and after surgery, nonetheless, Paired t-test was used. If the distribution of variables was not normal the Wilcoxon test was applied to compare with variables both before and after surgery. A Repeated Measure test was used to compare HRQoL three times (0, 6 and 12 months after surgery) P-value less than 0.05 was considered as a significant level of statistical tests.

## Results

**Fig. 1:**
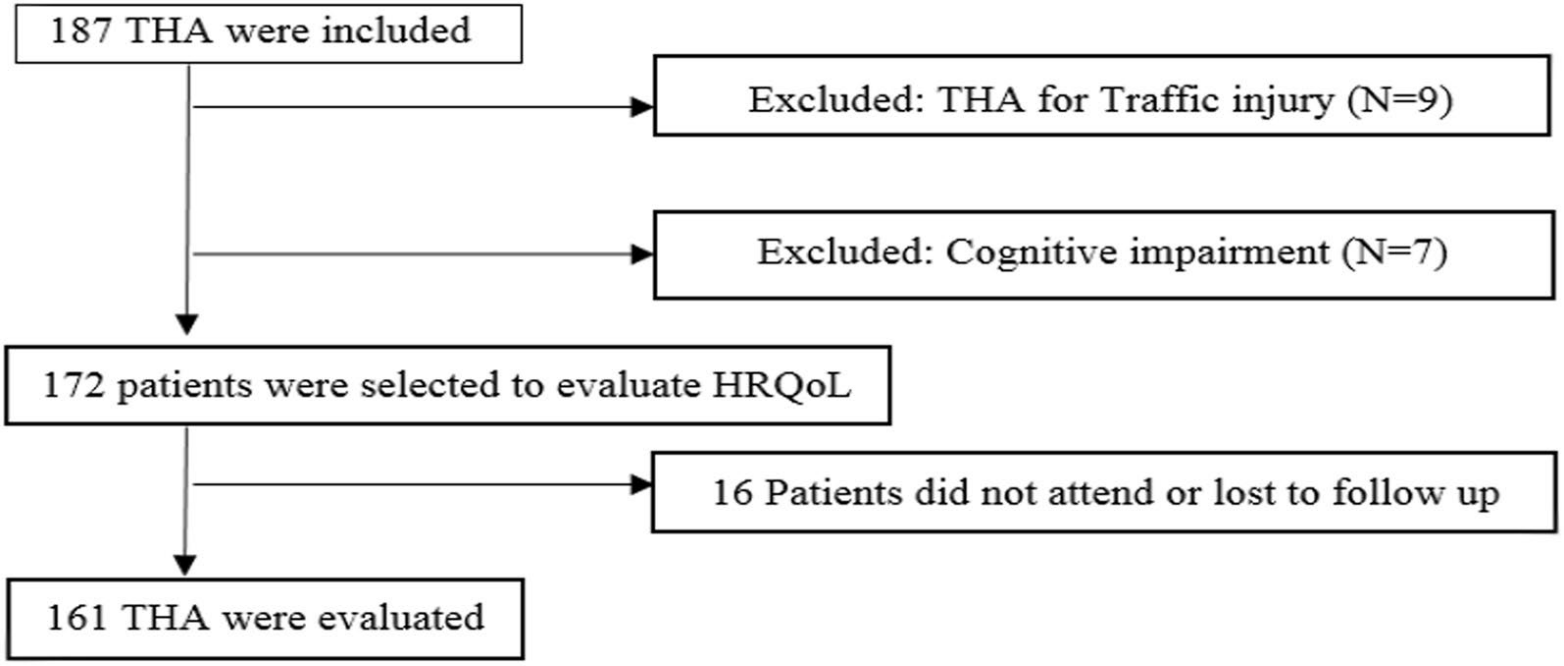
The flowchart of the study

Among 187 patients who have met the eligibility criteria and undergone THA surgery, 26 patients were excluded from the study. Nine patients had been done THA due to injuries caused by accidents. Six patients were excluded from the study in terms of the psychological disorders. Four patients did not fill in the consent form. Seven patients did not complete the follow-up period. Finally, 161 over 60 years old patients have completed the study, and their data are evaluated (Fig. 1).

### Demographic data

The overall mean age of patients was 69.1 ± 8.5 years (60 to 84 years). Most patients were female (73.9%). The mean BMI in the patients was 24.88 ± 4.2. 100 patients had upper diploma education. 119 patients (73.9%) did not smoke. At last, 107 of patients (66.5%) had more than two underlying diseases at the same time. The distribution of demographic variables is reported in Table 1.

**Table 1:** Demographic characteristics and number of comorbidities

Variable	(n = 161)
Age (year)	69.1 ± 8.55
**Gender**	
Female	119 (73.9)
Male	42(26.1)
**Educational status**	
Illiterate	17 (10.6)
Under diploma	44 (27.3)
Upper diploma	100 (62.1)
**Smoking history**	
Positive	42 (26.1)
Negative	119 (73.9)
BMI (kg/m^2^)	24.88 ± 4.2
Number of comorbidities	
< 2	54 (33.5)
≥ 2	107 (66.5)

BMI body mass index

### Comparison of results before THA and 6 months after THA

Six months after THA, except for the emotional role and vitality subscales, the other subscales were significantly higher than before THA (*P* < 0.05). The mean PCS score 6 months after THA was considerably higher than before surgery [(24.1 ± 11.2 vs 10.6 ± 9.8); (effect size: 0.97 and *P* = 0.023)]. There was no remarkable difference between the mean MCS scale before and 6 months after THA [(32.1 ± 18.4vs. 37.16 ± 21.5); (effect size: 0.16 and *P* = 0.067)] (Table 2).

**Table 2:** Comparison of the subscales of SF-36, PCS and MCS score before and 6 months after THA

Variable	Before THA	6 months after THA	Effect size (Cohen’s d)	*P* value*
**Subscales (Mean ± SD)**				
Physical performance	10.54 ± 8.5	22.83 ± 9.7	0.93	0.028
Physical role	10.3 ± 9.3	28.5 ± 12.5	1.21	0.001
Mental health	12.4 ± 28.2	33.5 ± 21.2	0.61	0.028
Vitality	25.6 ± 19.4	37.8 ± 26.8	0.28	0.059
Emotional role	42.6 ± 16.6	54.4 ± 25.7	0.32	0.061
Social role	8.6 ± 7.5	25.3 ± 12.5	0.96	0.011
Physical pain	10.03 ± 7.3	40.5 ± 13.3	1.57	0.001
General health	16.3 ± 8.1	35.5 ± 14.1	0.96	0.003
MCS	32.1 ± 18.4	37.16 ± 21.5	0.16	0.067
PCS	10.6 ± 9.88	24.1 ± 11.2	0.97	0.023

MCS, mental component score; PCS, physical component score; THA, total hip arthroplasty

*P value fewer than 0.05 is considered significant

### Comparison of results before THA and 12 months after THA

Twelve months after THA, the mean score in all SF-36 subscales were significantly higher than before surgery (*P* < 0.05). The mean PCS score was notably higher 12 months after THA than before THA [(41.2 ± 11.5 vs. 10.6 ± 9.90); (effect size: 1.89 and *P* = 0.012)]. However, 12 months after THA, the mean MCS score was noticeably higher than before THA. [(55.6 ± 22.3 vs 32.1 ± 18.4); (effect size: 0.86 and *P* = 0.048)] (Table 3).

**Table 3:** Comparison of the subscales of SF-36, PCS, and MCS score before and 12 months after THA

Variable	Before THA	12 months after THA	Effect size (Cohen’s d)	*P* value*
**Subscales (Mean ± SD)**				
Physical performance	10.54 ± 8.5	35.11 ± 10.5	1.61	0.001
Physical role	10.3 ± 9.3	38.6 ± 11.4	1.69	0.001
Mental health	12.4 ± 28.2	45.5 ± 22.1	1.01	0.008
Vitality	19.6 ± 13.4	59.4 ± 20.5	1.46	0.022
Emotional role	42.6 ± 13.6	75.4 ± 24.6	1.19	0.042
Social role	8.6 ± 7.5	32.5 ± 13.2	1.38	0.001
Physical pain	10.03 ± 8.3	56.3 ± 14.3	1.35	0.001
General health	16.3 ± 8.1	50.5 ± 16.5	1.51	0.001
MCS	32.1 ± 18.4	55.6 ± 22.3	0.88	0.048
PCS	10.6 ± 9.9	41.2 ± 11.5	1.89	0.012

MCS, mental component score; PCS, physical component score; THA, total hip arthroplasty

*P value fewer than 0.05 is considered significant

### Comparison of results 6 and 12 months after THA

The mean score for the emotional role subscale was substantially higher 12 months after THA surgery than 6 months after surgery (effect size: 0.82 and *P* = 0.031), according to subscale comparison findings. Moreover, the mean score for the vitality subscale 12 months after THA surgery improved significantly compared to 6 months after surgery (effect size: 0.73 and *P* = 0.048). In contrast, no appreciable difference was observed for the mean score of other subscales at 6 and 12 months after THA (*P* > 0.05). Thus, no conspicuous difference was reported for MCS and PCS subscales at 6 and 12 months after THA (*P* > 0.05).

## Discussion

This study aimed to compare HRQoL before and After THA in elderly people. The results of the present study showed that a half and a year after THA, HRQoL noticeably increased, especially measured against before THA.

SF-36 questionnaire was drawn on to assess HRQoL. SF-36 is a 36-item health questionnaire which was shown to be effective in more than 130 conditions and diseases [26]. The questionnaire consists of eight subscales, each of which has measured different aspects of health [15] and was summarized in two independent summary measures, including PCS and MCS, which assessed physical and mental health, respectively [27, 28]. The eight subscales provided a comprehensive profile of health status, but two summary measures (PCS and MCS) had unique advantages, including greater accuracy, lower ceiling and floor effect (an important factor in statistical analysis), and simpler analysis [27, 29].

PCS score went up notably in the first 6 months after THA compared to before THA, but the MCS score did not differ. After 12 months, MCS score ameliorated considerably compared to before THA. The interpretation of these results indicated in the first 6 months after THA, the promotion in the physical aspect of HRQoL (PCS) was responsible to improve HRQoL in the elderly and in the following (12 months after THA) promotion in the mental aspect (MCS) in addition to the physical aspect, was responsible for improving HRQoL after surgery. These changes in effect size were also evident. The effect size for MCS after 12 months (0.97) significantly increased compared to 6 months (0.19) after surgery and changed from poor to excellent. In fact, Cohen’s d showed that 6 months after the THA, the recovery rate for MCS was poor, which was reported to be excellent after 12 months.

Because the hip joint dysfunction caused pain in THA candidate patients, controlling and tranquilizing pain in terms of the anatomical corrections following THA was the first obvious change in the patient [30]. Therefore, in the primary follow-up after THA, the highest improvement among SF-36 subscales was expected to belong to the physical pain. The results of our study also showed that the physical pain subscale had ameliorated approximately four times drew an analogy between before and after THA, which none of the subscales surprisingly has improved this much. As a result of THA which led to improve physical function in daily life, moreover, following anatomical correction and pain control and the ability to walk painlessly [30, 31]. Therefore, after the physical pain subscale, it was expected that the highest amelioration in SF-36 subscales would be related to the physical and social role subscales. Our study shows this procedure to the largest augment among subscales after physical pain was in accord with physical and social role subscales. According to these explanations, a significant increase in PCS score in the first 6 months of follow-up could be related to the early positive effects of THA on pain control followed by painless walking. In other words, in the first 6 months after THA, the patient felt the positive effects of the surgery more on her/his physical aspect (than the psychological aspect). The effect size was reported to be good for PCS 6 months and 12 months after surgery (0.86 VS 1.89). In other words, surgery completely improved this scale after 6 months, which increased over time and approached the level of function in normal people for the elderly. During the next 6 months of follow-up after THA, improvement in both the psychological state and the daily life of patient was evident. Thus, in the 12 months after THA, the MCS score (which in the first 6 months of follow-up was not significantly different from before surgery) augmented noticeably measured against before THA.

Regarding the remarkable growth in the number of THA surgeries worldwide, many research have climbed an upward trend concerning this field. In 2020, Miettinen et al., conducted a retrospective single-center study HRQoL drawing an analogy between before and after THA and total knee arthroplasty (TKA) in Finland. The results of their study showed that HRQoL significantly improved after THA compared to before surgery. They have considered painless movement as a possible cause of a significant increase in postoperative HRQoL [31]. Although, the results of our study (the improvement of HRQoL after THA) were in accord with the results of their study, there were differences in how to form the study. Firstly, Miettinen et al., used the 15D questionnaire to assess HRQoL, while we have used the SF-36. Secondly, made a distinction between with post-THA HRQoL and with pre-THA; furthermore, they compared post-THA HRQoL with normal 15D values in the general population and showed that in individuals over 75 years of age, though this post-THA value was equal to the normal 15D score. No such comparison was made in our study. Finally, two domains of HRQoL, including PCS and MCS, were examined and compared separately in our study, whereas this comparison was not drawn in the study of Miettinen et al. In 2019, Luo et al., conducted a study to evaluate HRQoL after THA in the patients with childhood hip infections. In the following, the mean age of patients was 52.3 years and the mean duration of follow-up was 6.1 years. Luo et al., was similar to our study, in order to recording the HRQoL score, two summary measures including PCS and MCS were also assessed and recorded. But unlike our study, these two outcomes (PCS and MCS) were recorded only once (final follow-up, mean of 6.1 years after surgery, range 2.1–9.6 years). In accord with this study, the results of respected study have expressed an appreciable rise in PCS and MCS after THA in HRQoL, [32]. Because Luo et al., did not perform two follow-ups like this study (6 and 12 months after THA), the results of that study, especially for PCS and MCS, were not comparable to the results of our study. Regarding only one follow-up, it was impossible to differentiate which aspect of HRQoL (either PCS or MCS) was consistent with the change in HRQoL in the short and long term. In 2011, Mariconda et al., has followed up HRQoL after THA. Consequently, they concluded that the patients with hip joint disorders requiring THA, who did THA (case group), had a lower SF-36 physical indexes (physical aspect of HRQoL) and hip functionality (according to Harris Hip and WOMAC scores) after surgery than normative values but had better SF-36 physical indexes and hip functionality than similar individuals who did not do THA (control group) [33]. They reported a very high level of postoperative satisfaction of the patients and stated that this high level of satisfaction did not contradict the low values of physical indexes of quality of life in these patients, because post-THA satisfaction did not depend only on physical indexes (such as a hip function). Improvements in pain index and psychological status have played a prominent role in post-THA satisfaction [34].

A 2019 study by Bahardost et al., showed that HRQoL in the case group was significantly lower than the control group after 27 months of follow-up [10]. In the study of Bahardost et al., Perhaps, if HRQoL of postoperative patients were compared with HRQoL before surgery, the results could have been similar to our study. The type of nation was one of the factors that influenced the findings of such investigations. Because the welfare, financial, and HRQoL in developed nations were substantially greater, HRQoL studies in developed countries were considerably more positive than research in developing countries [35].This study was conducted in a developing country. On the other hand, this variable could have affected its results. In respect of this study represented a comparative basis for similar studies designed in developed countries. In 2016, Sanei et al., made a distinction between before and after THA in Iran. On the contrary to this study, Sanei et al. have included the middle-aged population (mean age 43.89 years) and performed only one follow-up (6 months after THA). They did not study PCS and MCS. In the study of Sanei et al., as in our study, HRQoL after 6 months has significantly increased and the highest improvement (nearly 11.7 times more) was seen in the pain subscale (bodily pain) [21]. In 2015, Shahcheraghi et al., conducted a retrospective study to compare HRQoL results following THA using two types of prostheses. Thus, Shahcheraghi study was similar to the study of Sanei et al., only one follow-up (mean of follow-up: 65 months, range 26–136 months) was performed in the middle-aged population (mean age 48.83 years), however, PCS and MCS were not studied [36].

The prospective nature of the study, conducting the study in the elderly population, and the interpretation of the mental (MCS) and physical (PCS) aspects of HRQoL are the main strengths of the present study. One of the considerable points in this study was the lion’s share of the patients which included women (73.9%). Because the factors affecting the quality of life of men and women were different [37]. Consequently, it was impossible to generalize these results by gender because our study was not designed to separate gender. Therefore, it was recommended that this field of study should have designed by gender in the future. Among the limitations of the present study were the small sample size. Because the burden of hospitalization affected the outcomes of the study [38], one of the limitations of this study could be the inclusion of patients from high-volume arthroplasty treatment centers. Although the surgical method was similar in high or low-volume arthroplasty treatment centers, the quality of the surgery was affected by the number of surgeries performed per day. Therefore, the results of this study hardly could have generalized to low-volume arthroplasty centers. It was suggested that the future studies in this field would have been conducted in a multi-center manner to generalize the results to all medical centers.

## Conclusion

From the follow-up of 12 months after THA surgery in the elderly who need this operation, it can be concluded that this surgery significantly improves HRQoL. Improvement in HRQoL in the first 6 months after THA is related to promotion in the physical aspect (PCS score), and in the second 6 months after THA is related to promotion in the psychological aspect (MCS score).

## Supplementary Information

**Additional file 1**. SF-36 questionnaire.

## Data Availability

Given that the data of this study is a small part of the data of a large study and according to the forecast of a series of articles will be extracted serially from this data. Data will not be available until the end of the project, although data-sets used and analyzed during the current study are available from the corresponding author upon reasonable request.

## Abbreviations

BMI: Body mass index
HRQoL: Health-related quality of life
MCS: Mental component score
PCS: Physical component score
SF-36: Short Form 36 health survey
THA: Total hip arthroplasty
